# The C5a/C5aR1 axis controls the development of experimental allergic asthma independent of LysM-expressing pulmonary immune cells

**DOI:** 10.1371/journal.pone.0184956

**Published:** 2017-09-20

**Authors:** Anna V. Wiese, Fanny Ender, Katharina M. Quell, Konstantina Antoniou, Tillman Vollbrandt, Peter König, Jörg Köhl, Yves Laumonnier

**Affiliations:** 1 Institute for Systemic Inflammation Research, University of Lübeck, Lübeck, Germany; 2 Cell Analysis Core, University of Lübeck, Lübeck, Germany; 3 Institute for Anatomy, University of Lübeck, Lübeck, Germany; 4 Division of Immunobiology, Cincinnati Children’s Hospital Medical Center and University of Cincinnati College of Medicine, Cincinnati, Ohio, United States of America; Universidade Federal do Rio de Janeiro, BRAZIL

## Abstract

C5a regulates the development of maladaptive immune responses in allergic asthma mainly through the activation of C5a receptor 1 (C5aR1). Yet, the cell types and the mechanisms underlying this regulation are ill-defined. Recently, we described increased C5aR1 expression in lung tissue eosinophils but decreased expression in airway and pulmonary macrophages as well as in pulmonary CD11b^+^ conventional dendritic cells (cDCs) and monocyte-derived DCs (moDCs) during the allergic effector phase using a floxed green fluorescent protein (GFP)-C5aR1 knock-in mouse. Here, we determined the role of C5aR1 signaling in neutrophils, moDCs and macrophages for the pulmonary recruitment of such cells and the importance of C5aR1-mediated activation of LysM-expressing cells for the development of allergic asthma. We used LysM-C5aR1 KO mice with a specific deletion of C5aR1 in LysMCre-expressing cells and confirmed the specific deletion of C5aR1 in neutrophils, macrophages and moDCs in the airways and/or the lung tissue. We found that alveolar macrophage numbers were significantly increased in LysM-C5aR1 KO mice. Induction of ovalbumin (OVA)-driven experimental allergic asthma in GFP-C5aR1^fl/fl^ and LysM-C5aR1 KO mice resulted in strong but similar airway resistance, mucus production and Th2/Th17 cytokine production. In contrast, the number of airway but not of pulmonary neutrophils was lower in LysM-C5aR1 KO as compared with GFP-C5aR1^fl/fl^ mice. The recruitment of macrophages, cDCs, moDCs, T cells and type 2 innate lymphoid cells was not altered in LysM-C5aR1 KO mice. Our findings demonstrate that C5aR1 is critical for steady state control of alveolar macrophage numbers and the transition of neutrophils from the lung into the airways in OVA-driven allergic asthma. However, C5aR1 activation of LysM-expressing cells plays a surprisingly minor role in the recruitment and activation of such cells and the development of the allergic phenotype in OVA-driven experimental allergic asthma.

## Introduction

Allergic asthma is a chronic pulmonary disease which manifests as an inappropriate immune response to aeroallergens in susceptible individuals. Allergic asthma is characterized by a Th2/Th17 maladaptive immune response. In the last decades, the anaphylatoxins C3a and C5a and their cognate receptors have been recognized as important regulators of the development of the disease [1]. In particular, C5a exerts dual functions during the sensitization and the effector phase of allergic asthma [1, 2]. Pharmacological targeting of C5aR1 during the sensitization phase increases the severity of the asthmatic phenotype, while targeting of C5aR1 during the effector phase reduces the allergic asthma phenotype [2, 3]. In addition, the C5a/C5aR1 signaling axis has been identified as a main regulator of dendritic cell (DC) functions and the development of maladaptive Th2/Th17 immune responses [4, 5]. Furthermore, pDCs can suppress myeloid dendritic cell functions by a C5aR1-dependent mechanism [2, 6]. More recent studies have broadened our understanding regarding the role of C5aR1 in DC functions. Adoptive transfer of C5aR1^-/-^ bone marrow derived (BM)DCs demonstrated that C5aR1 controls the differentiation of myeloid-derived suppressor cells from BM cells thereby suppressing DC-dependent T cell proliferation and differentiation [7]. Further, a recent study using a GFP-C5aR1 knock-in mouse demonstrated that C5aR1 expression is regulated in several innate immune cells that play important roles for the development of the allergic phenotype during the effector phase. More specifically, C5aR1 expression was downregulated in airway and tissue alveolar macrophages, CD11b^+^ conventional (c)DCs and monocyte-derived (mo)DCs but upregulated in eosinophils in an OVA-induced allergic asthma experimental model using GFP-C5aR1^fl/fl^ mice [8].

In addition to DCs, three cell populations express C5aR1 in the lungs at steady state, i.e. airway and tissue alveolar macrophages (AMs), eosinophils and neutrophils [8, 9]. So far, no role for C5aR1 has been reported for eosinophil activation in allergic asthma, although C5a is a potent chemoattractant and activator of eosinophils [10, 11]. Furthermore, C5a increases the adhesion of eosinophils through upregulated expression of CD11b [12]. Further, C5aR1 regulates macrophages functions. For example, C5a suppresses TLR-induced IL-12 family cytokine production but promotes and enhances IL-6 production from macrophages [13–15]. However, alveolar macrophages are an atypical macrophage population which strongly expresses CD11c and SiglecF [16] but lacks the expression of C3aR [17]. In alveolar macrophages, C5aR1 has been reported in a lung Arthus reaction model [18]. Similarly, C5aR1 is a well-known regulator of neutrophil functions [19]. However, its role in resident pulmonary and inflammatory neutrophil regulation during allergic asthma is ill-defined.

Until recently, tools were lacking to determine *in situ* functions of C5aR1 in specific pulmonary cell types in allergic asthma models. To close this gap, we generated LysM-C5aR1 KO mice by crossing GFP-C5aR1^fl/fl^ knock-in with LysMCre mice [9]. In LysM-C5aR1 KO mice, we observed the expected conditional C5aR1 deletion in LysM-expressing cells, such as neutrophils and macrophages rendering them insensitive to C5a activation [9]. Here, we used C5aR1^fl/fl^ and LysM-C5aR1 KO mice in a model of OVA-driven experimental allergic asthma to assess the impact of conditional C5aR1 deletion inLysM-expressing cells on their recruitment to the airways and the lung tissue and the development of allergic asthma.

## Materials and methods

### Mice

GFP-C5aR1^fl/fl^ and LysM-C5aR1 KO were described previously [9]. All mice were bred and maintained at the University of Lübeck specific pathogen-free facility and used for experiments at 8–12 weeks of age. Animal care was provided in accordance with German rights. This study was reviewed and approved by the Schleswig-Holstein state authorities (Nr. V242-30397/2016 (56-5/16)).

### Experimental ovalbumin (OVA)-driven allergic asthma model

The OVA-induced asthma model was performed as described previously [8]. Briefly, mice were immunized by intra-peritoneal (i.p.) injection with OVA-Alum (200μg/2mg) on days 0 and 7 and challenged intratracheally (i.t.) with 750 μg OVA (in 50 μl PBS) on days 14, 16, 18, and 20. On day 21, 24 h after the last i.t. administration, airway hyperresponsiveness (AHR) was determined and tissue samples were harvested for further analysis.

### Determination of airway hyperresponsiveness

Mice were anaesthetized by i.p. injection of 50 μl Ketavet/Rompun (190 mg/kg bodyweight/ 12 mg/kg bodyweight respectively, Pfizer/Bayer). Muscle relaxation was induced by administration of 50 μl of Esmeron (25 mg/kg bodyweight, Organon). AHR was measured in anesthetized mice that were mechanically ventilated using a FlexiVent (SciReq) system as described [7, 20]. Aerosolized acetyl-β-methyl-choline (methacholine) (0, 2.5, 5, 10, 25, and 50 mg/ml; Sigma-Aldrich) was generated by an ultrasonic nebulizer and delivered in-line through the inhalation port for 10 seconds. Airway resistance was measured two minutes later.

### Collection of bronchoalveolar lavage (BAL) fluid and differential cell counting

BAL fluid samples were obtained by cannulating the trachea, injecting 1 ml of ice-cold PBS, and by subsequently aspirating the BAL fluid. Then, the volume of the aspirated BAL fluid was recorded. After red blood cell lysis, BAL fluid cells were washed once in PBS and counted using a Neubauer chamber (Assistant, Germany), and calculated as cells/ml based on the cell count and the volume of the aspirated BAL fluid. Frequencies of BAL fluid cells were determined by flow cytometry. Cell numbers of defined BAL cell populations were calculated using the frequency of such cells, as determined by flow cytometry, and the total BAL cell number per ml.

### Antibodies

Monoclonal phycoerythrin (PE)-labeled Ab against C5aR1/CD88 (20/70) was obtained from AbDSerotec. Brilliant Violet (BV) 421- or PE-labeled Abs against SiglecF (E50-2440), V450- or allophycocyanin (APC)-labeled Abs against Ly6G (1A8) were purchased from BD Biosciences. EFluor (eF)450-labeled Abs against CD19 (1D3), CD3e (145-2C11) or CD49b (DX5), APC-labeled Ab against CD11c (N418) and PE-cyanine (Cy)7-labeled Ab against CD4 (RM4-5) were obtained from eBioscience (Affimetrix). BV510-labeled Ab against CD11b (M1/70), PE-labeled Ab against CD64 (X54-5/7.1), APC-eF780–labeled Ab against MHC class II (M5/144.15.2), APC-labeled Ab against CD62L (MEL-14), BV421-labeled Ab against CD44 (IM7), and Peridinin chlorophyll protein–cyanine 5.5 (Per-CP–Cy5.5)–labeled Ab against CD103 (2E7) were all purchased from Biolegend. Per-CP–Cy5.5–labeled Ab against CD3 (17A2), CD5 (53–73), CD27 (LG.7F9), NK1.1 (PK136), TCRβ(H57-597), CD11b (MI/70); eF780–labeled Ab against CD11c (N418), B220 (RA3-6B2), CD49b (DX5); eF450–labeled Ab against CD25 (PC61.5), PE-labeled Ab against CD90.2 (30-H12), PE-cy5-labeled Ab against CD127 (A7R34) were purchased from eBioscience (Affimetrix) except anti-CD90.2 (BD Pharmingen).

### Lung cell isolation and flow cytometric analysis

Liberase TL (Roche) 0.25 mg/ml and DNaseI 0.5 mg/ml (Sigma-Aldrich) digests of the lungs were prepared to obtain single lung cell suspensions. Phenotypic characterization of cells was performed on a BD LSRII or an ARIA III flow cytometer using recently published gating strategies [8, 9, 16]. ILC2s were identified as Lineage− cells lacking the expression of lineage markers associated with T cells (CD3, CD5, TCR and CD27), B cells (B220), macrophages (CD11b), DCs (CD11c) and natural killer (NK) cells (NK1.1, CD49b). These Lineage− cells expressed CD90.2 (alloantigen Thy-1), CD25 (α-chain of the receptor for IL-2) and CD127 (α-chain of the receptor for IL-7) [21].

### Lung histology

Lung histological staining, detection and quantification of mucus cell content were done as described previously [22]. Serial sections of lungs from PBS- or OVA-treated mice were stained either with hematoxylin and eosin (H/E), or periodic acid-Schiff (PAS). Airways of four lung sections were counted by light microscopy. To evaluate the frequency of mucus producing airways in the different groups, PAS positive airway frequencies were calculated as the percentage of number of PAS positive airways relative to the total number of airways.

### RNA isolation from CD4^+^CD44^+^CD62L^-^ T effector cells and real time PCR

CD4^+^CD44^+^CD62L^-^ effector T cells were sorted using a BD FACS ARIA III. RNA was isolated using Trizol reagent according to the manufacturer’s instructions (Invitrogen). Reverse transcription reaction of total RNA was performed after degradation of contaminating genomic DNA using DNase I (Fermentas), using a first strand cDNA synthesis kit (Revertaid Premium, Fermentas). Quantitative PCR was done using iQSyber green (Biorad) on a CFX96 real-time PCR system (Biorad) using the specific primers (Eurofin) as previously described [8]. Differentiation of T helper (Th) cells was evaluated by amplifying Th-specific transcription factor mRNAs: *TBX21* (Th1) 5’-GGTGTCTGGGAAGCTGAGAG-3’ (sense) and 5’-ATCCTGTAATGGCTTGTGGG-3’ (antisense), *GATA3* (Th2) 5’-GCCTGCGGACTCTACCATAA-3’ (sense) and 5’-AGGA TGTCCCTGCTCTCCTT-3’ (antisense), *RORγT* (Th17) 5’-CCGCTGAGAGGGCTTCAC-3’ (sense) and 5’-TGCAGGA GTAGGCCACATTACA-3’ (antisense) and *FoxP3* (Treg) 5’-CCCATCCCCAGGAGTCTTG-3’ (sense) and 5’-ACCATGACTAGGGGCACTGTA-3’ (antisense). To determine the cytokine expression, we amplified the mRNA for *IL-13* using 5’-CCTGGCTCTTGCTTGCCTT-3’ (sense) and 5’- GGTCTTGTGTGATGTTGCTCA-3’ (antisense) primers, and *IL-17A* using 5’- CTCCAGAAGGCCCTCAGACTAC-3’ (sense) and 5’- AGCTTTCCCTCCGCATTGACACAG-3’ (antisense) primers. In all samples, *actin* mRNA was amplified using the primers 5’-GCACCACACCTTCTACAATGAG-3’ (sense) and 5’-AAATAGCACAGCCTGGATAGCAAC-3’ (antisense) and used as an internal control. Real-time RT-PCR data were analyzed using CFX Manager Software 3.1 (Bio-Rad).

### Statistical analysis

Statistical analysis was performed using the GraphPad Prism version 5 (GraphPad Software, Inc.). Normal distribution of data was tested using the Kolmogorov-Smirnov or D'Agostino-Pearson tests, some after log transformation. When groups were normally distributed, statistical differences between two groups were analyzed by unpaired t test. Comparisons involving multiple groups were first analyzed by ANOVA followed by Tukey's test. When groups were not normally distributed, they were analyzed using a Mann-Whitney U (two groups), or an ANOVA on ranks (mutiple groups) followed by a Dunn's multiple comparison test. A p value < 0.05 was considered significant. *p<0.05, ** p <0.01 and ***p <0.001.

## Results

### GFP-C5aR1 is conditionally deleted in pulmonary neutrophils, macrophages and DCs but not in eosinophils

**Fig 1:**
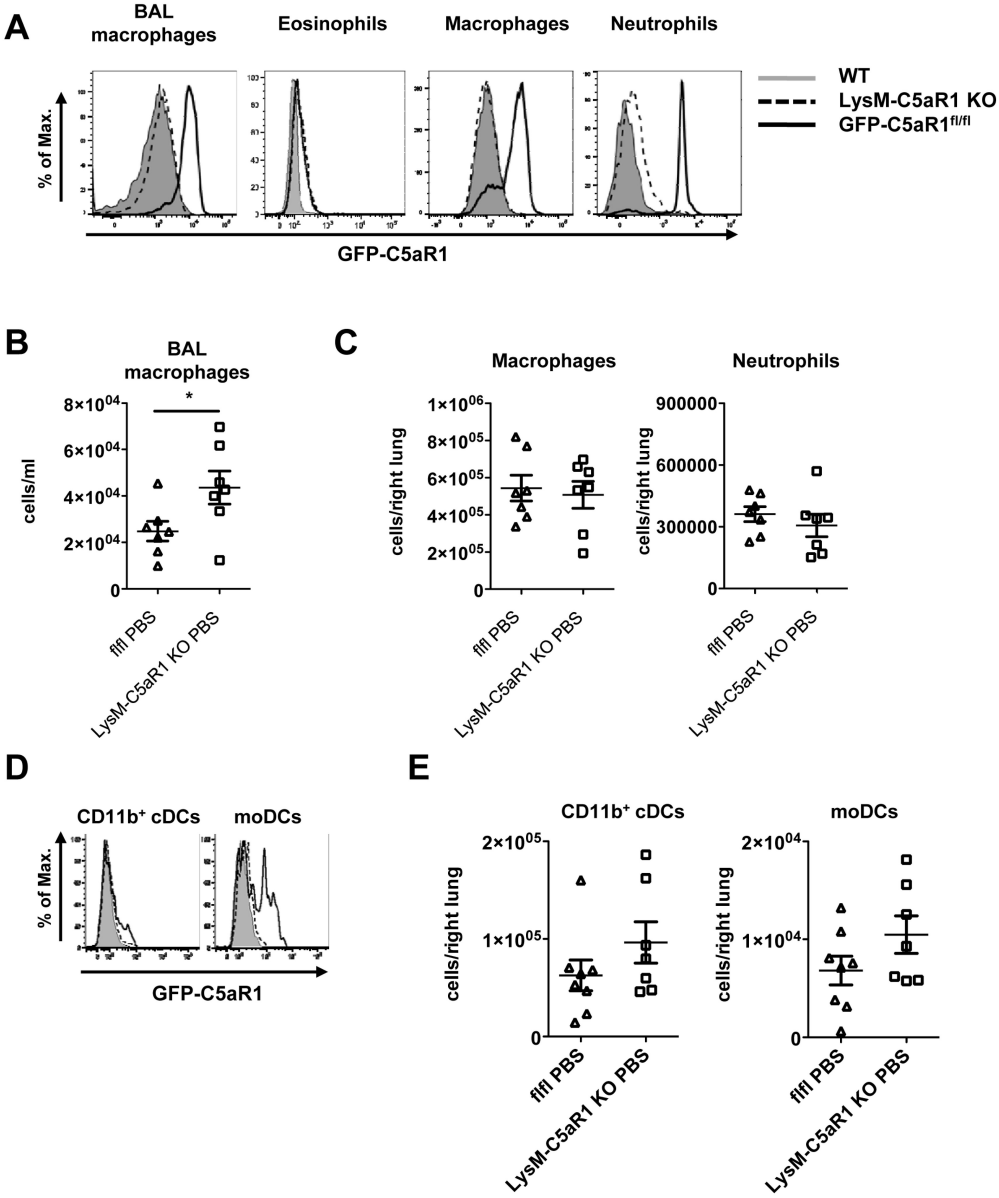
C5aR1 expression in pulmonary cells. **(A)** GFP-C5aR1 expression in BAL alveolar macrophages, lung eosinophils, macrophages, and neutrophils of GFP-C5aR1^fl/fl^ or LysM-C5aR1 KO mice. Grey histogram: GFP signal in WT cells; dashed line: GFP signal in cells from LysM-C5aR1 KO mice; solid line: GFP signal in cells from GFP-C5aR1^fl/fl^ mice. Data are representative of at least 3 independent experiments. **(B)** Scatter plot showing the number of AMs present at steady state in the airways of GFP-C5aR1^fl/fl^ (open triangle) or LysM-C5aR1 KO mice (open square). **(C)** Scatter plot showing the number of tissue-associated AMs and neutrophils in GFP-C5aR1^fl/fl^ (open triangle) or LysM-C5aR1 KO mice (open square). **(D)** GFP-C5aR1 expression in lung DCs of GFP-C5aR1^fl/fl^ or LysM-C5aR1 KO mice. Grey histogram: GFP signal in WT cells; dashed line: GFP signal in cells from LysM-C5aR1 KO mice; solid line: GFP signal in cells from GFP-C5aR1^fl/fl^ mice. Data are representative of at least 3 independent experiments. **(E)** Scatter plot showing the number of DC subsets present at steady state in the lungs of GFP-C5aR1^fl/fl^ (open triangle) or LysM-C5aR1 KO (open square) mice. For all plots, values shown are the mean ± SEM; n = 7 per group. The asterisk indicates significant differences between PBS-treated GFP-C5aR1^fl/fl^ and LysM-C5aR1 KO mice. *p<0.05.

The *lys2* gene, encoding for the lysozyme (Lys)M protein, is expressed specifically by neutrophils and macrophages [23, 24]. In agreement, the expression of C5aR1 were abrogated in BM-neutrophils and peritoneal macrophages from LysM-C5aR1 KO mice [9]. Furthermore, we observed that, at steady state, LysM-C5aR1 KO airway and tissue-associated AMs, but not eosinophils were negative for GFP (Fig 1A), indicating that LysM^+^ pulmonary cells lost the expression of C5aR1. The GFP signal in pulmonary neutrophils of LysM-C5aR1 KO mice was low and similar to what we had observed in BM neutrophils [9]. Furthermore, we noticed that the absence of C5aR1 in AMs resulted in a small but significant increase in the number of AMs in the airways of LysM-C5aR1 KO compared to GFP-C5aR1^fl/fl^ mice (Fig 1B). In contrast, the number of tissue-associated AMs and neutrophils did not differ in the two strains (Fig 1C).

In addition, the two pulmonary DC populations reported to express GFP-C5aR1, CD11b^+^ cDCs and moDCs [8, 9], were negative for GFP-C5aR1 in the LysM-C5aR1 KO mice (Fig 1D), suggesting that these cell types also express LysM. However, no change in the number of DCs in lungs of GFP-C5aR1^fl/fl^ or LysM-C5aR1 KO mice could be observed (Fig 1E).

### Similar AHR, airway inflammation and mucus production in GFP-C5aR1^fl/fl^ and LysM-C5aR1 KO mice upon OVA-driven allergic asthma

**Fig 2:**
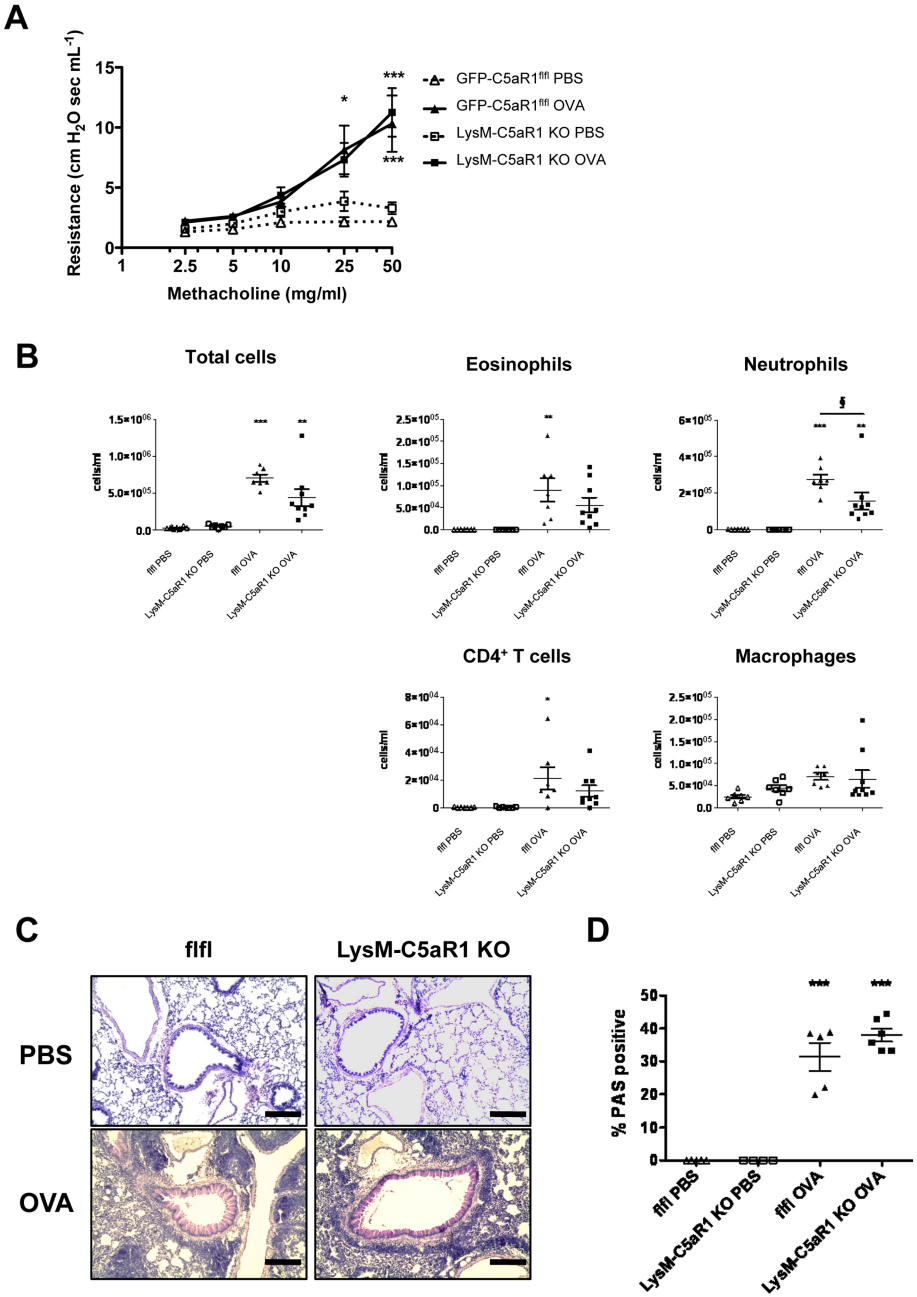
Deletion of C5aR1 in LysM-expressing cells controls recruitment of airway neutrophils but is dispensable for the development of strong AHR, airway inflammation and mucus production. **(A)** AHR in response to increased doses of nebulized methacholine in the airways, expressed as airway resistance. Shown are dose response curves in PBS-treated controls or OVA-immunized mice from the GFP-C5aR1^fl/fl^ and LysM-C5aR1 KO mice. Values shown are the mean ± SEM; n = 7–9 per group. **(B)** Total and differential cell counts in BAL fluid of GFP-C5aR1^fl/fl^ and LysM-C5aR1 KO mice in response to PBS treatment or OVA-immunization. Values shown are the mean ± SEM; n = 7–9 per group. **(C)** Histological examination of mucus production in airways of PBS-treated or OVA-immunized GFP-C5aR1^fl/fl^ or LysM-C5aR1 KO mice. Sections were stained with PAS for mucus production (original magnification x 200). Pictures shown are representative of n = 4–6 lungs per group. Scale bar represents 200μm. (**D)** Frequency of PAS-positive bronchi in PBS-treated or OVA immunized mice. Values shown are the mean ± SEM; n = 4–6 per group. Asterisks indicate significant differences between the PBS and OVA treatment groups, The § symbol indicates significant differences between OVA-treated GFP-C5aR1^fl/fl^ and LysM-C5aR1 KOmice. * or § p<0.05, ** p<0.01, and *** p <0.001.

Next, we determined the impact of C5aR1 deletion in LysM-expressing cells for the development of experimental OVA-driven allergic asthma. Upon OVA-induced allergic asthma, GFP-C5aR1^fl/fl^ and LysM-C5aR1 KO mice showed a significant increase in AHR in response to increasing concentrations of methacholine compared to PBS-treated control mice (Fig 2A). However, the methacholine dose response curves obtained in GFP-C5aR1^fl/fl^ and LysM-C5aR1 KO mice were indistinguishable.

The analysis of the BAL cell composition revealed a strong inflammatory cell influx into the alveolar space of GFP-C5aR1^fl/fl^ mice in OVA-treated mice as compared with the PBS controls, which was slightly lower in LysM-C5aR1 KO mice (Fig 2B). Neutrophils were the dominant cell population in OVA-challenged GFP-C5aR1^fl/fl^ and LysM-C5aR1 KO mice. Importantly, the number of airway neutrophils in LysM-C5aR1 KO mice was significantly lower than in GFP-C5aR1^fl/fl^ mice, suggesting that the C5a/C5aR1 axis controls the neutrophil recruitment into the airways. In both strains, the eosinophils showed a modest but similar increase upon allergic asthma. Also, the AM numbers did not differ between both strains suggesting that the absence of C5aR1 on macrophages is not critical for their recruitment into the alveolar space (Fig 2B). Finally, the recruitment of T cells was similar in both strains.

Next, the production of mucus by goblet cells in the different groups was evaluated (Fig 2C and 2D). Compared to PBS-treated control mice, GFP-C5aR1^fl/fl^ and LysM-C5aR1 KO mice showed a significant but similar increase in the frequency of mucus-positive airways upon OVA challenge (Fig 2D). In summary, deletion of C5aR1 in LysM-expressing cells results in significantly reduced airway neutrophilia, whereras eosinophilic and T cell airway inflammation is not altered. The reduced airway neutrophil number did not affect AHR or mucus production since GFP-C5aR1^fl/fl^ and LysM-C5aR1 KO mice suffered from comparable strong AHR and mucus production.

### The C5a/C5aR1 axis in LysM-expressing cells is dispensable for OVA-driven accumulation of pulmonary neutrophils, macrophages and DCs

**Fig 3:**
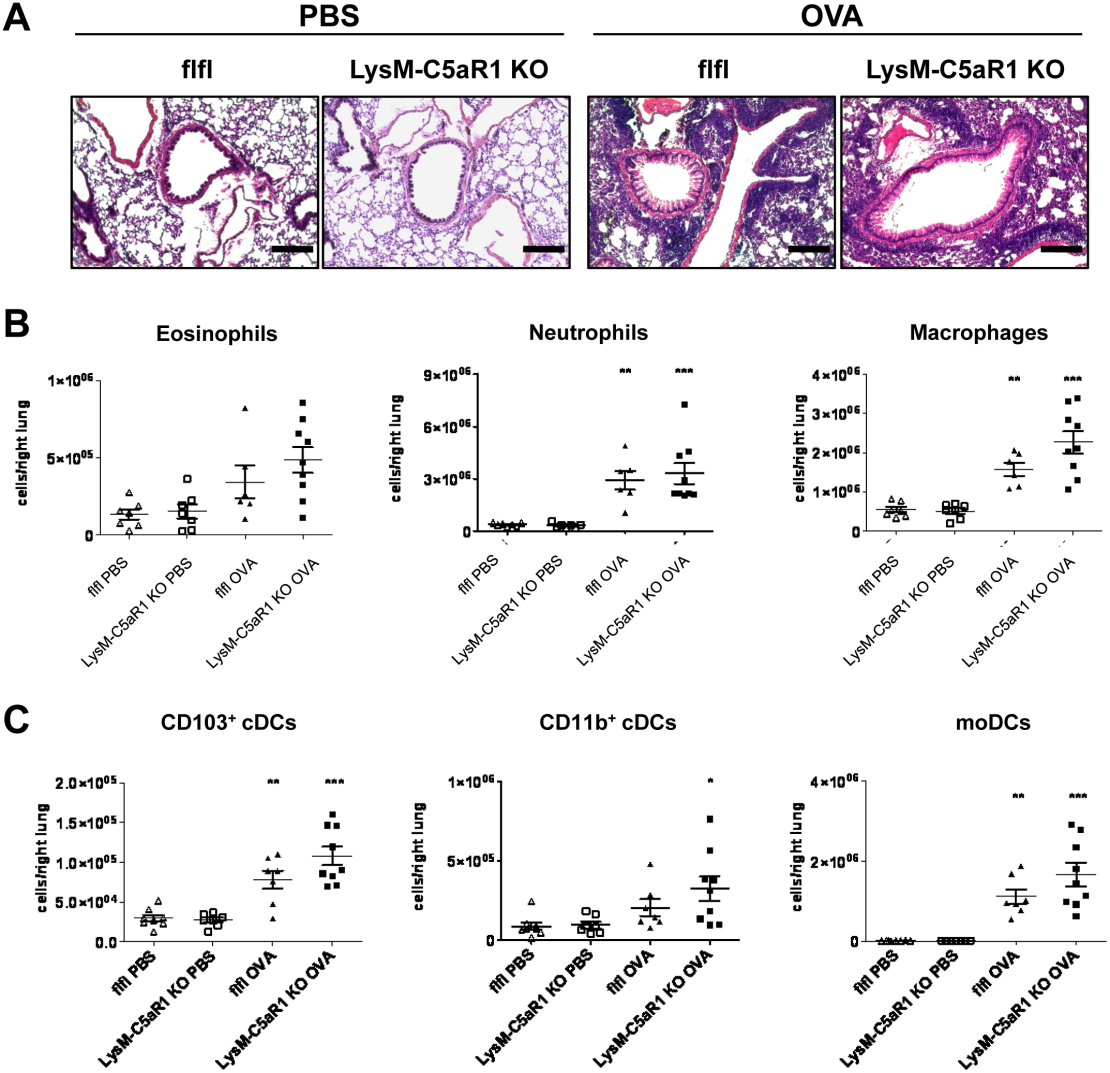
Deletion of C5aR1 in LysM-expressing cells does not affect the pulmonary accumulation of innate immune cells in response to OVA. **(A)** Histological examination of airway inflammation in GFP-C5aR1^fl/fl^ and LysM-C5aR1 KO mice. Sections from PBS or OVA-immunized mice were stained with H&E (original magnification x 200). Pictures shown are representative of n = 4–6 lungs per group. Scale bar represents 200μm. **(B)** Differential cell counts of lung eosinophils, neutrophils and macrophages of PBS-treated or OVA-immunized GFP-C5aR1^fl/fl^ or LysM-C5aR1 KO mice. Values shown are the mean ± SEM; n = 6–9 per group. **(C)** Cell counts of DC subsets in the lungs of PBS-treated or OVA-immunized GFP-C5aR1^fl/fl^ or LysM-C5aR1 KO mice. Values shown are the mean ± SEM; n = 6–9 per group. Asterisks indicate significant differences between the PBS and OVA treatment groups; * p<0.05, ** p<0.01, and *** p <0.001.

In the next step, we evaluated the pulmonary cell infiltration. Histologically, we found a strong recruitment of cells to the area between airways and blood vessels in response to OVA(Fig 3A).

Flow cytometric analysis showed that the number of eosinophils slightly increased after OVA challenge conditions (Fig 3B; left panel). Further, the neutrophil numbers in the lung significantly increased upon OVA challenge as compared to PBS controls (Fig 3B; middle panel). Similarly, macrophage numbers strongly increased in GFP-C5aR1^fl/fl^ and LysM-C5aR1 KO mice in response to repeated OVA administration as compared to PBS controls (Fig 3B; right panel). However, when we compared the number of cells in the GFP-C5aR1^fl/fl^ and LysM-C5aR1 KO mice, we found no differences in the recruitment of eosinophils, macrophages or neutrophils in OVA-driven allergic asthma conditions.

In line with the increase of innate inflammatory cells upon OVA challenge, CD103^+^ cDCs markedly increased in the lungs of GFP-C5aR1^fl/fl^ and LysM-C5aR1 KO mice in response to OVA (Fig 3C; left panel). CD11b^+^ cDCs increased only modestly after OVA challenge (Fig 3C; middle panel). MoDCs, which were almost completely absent in PBS-treated mice, were massively recruited into the lung upon OVA challenge (Fig 3C; right panel). We observed no differences in the recruitment of the DC subsets after OVA administration between GFP-C5aR1^fl/fl^ and LysM-C5aR1 KO mice. Thus, C5aR1 deletion in LysM-expressing cells does not affect the recruitment of pulmonary neutrophils, macrophages, CD11b^+^ or CD103^+^ cDCs as well as moDCs in OVA-driven allergic asthma.

### C5aR1 signaling in LysM-expressing cells has no impact on pulmonary accumulation and skewing of CD4^+^ T cells

**Fig 4:**
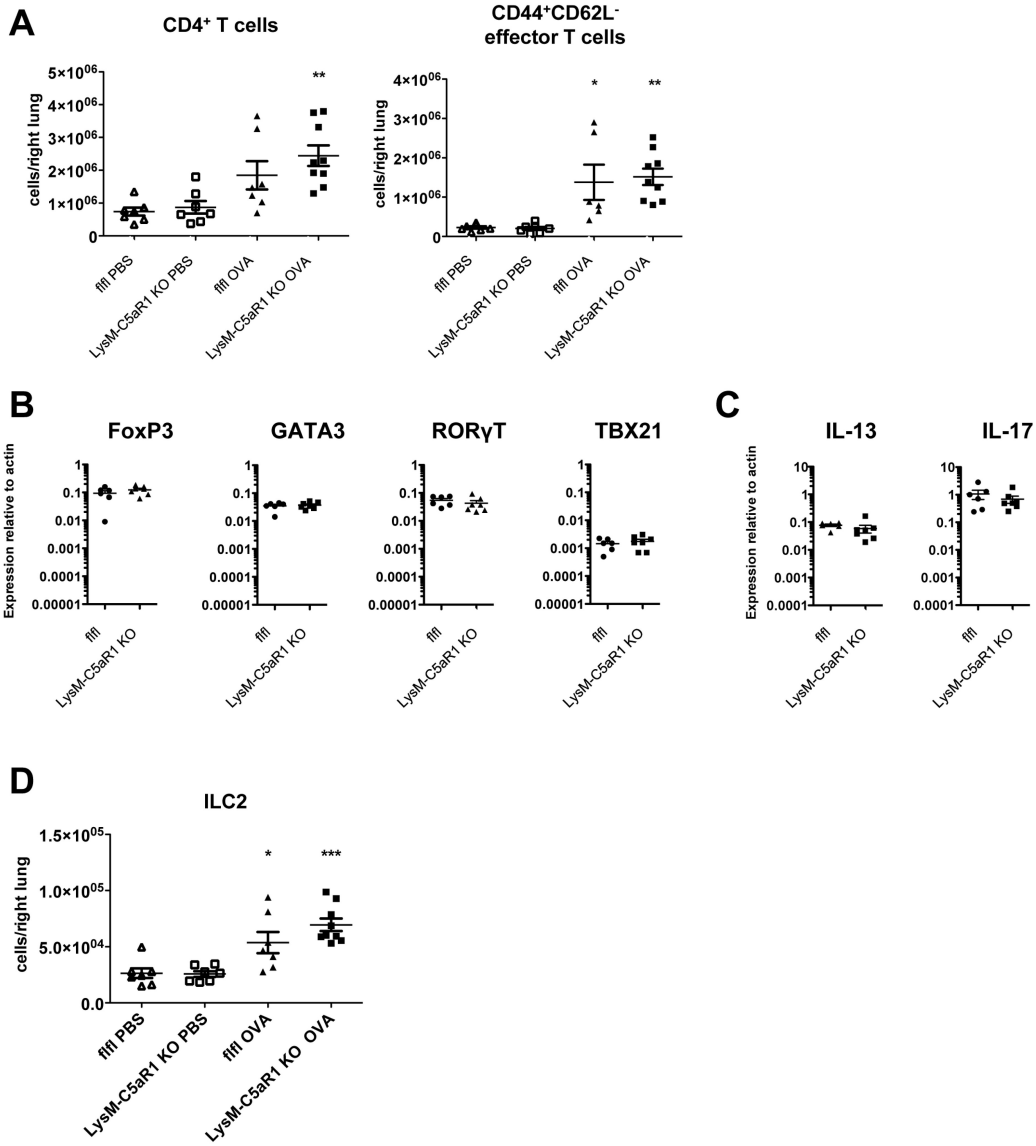
Similar accumulation of pulmonary CD4^+^ T and ILC2 in GFP-C5aR1^fl/fl^ and LysM-C5aR1 KO mice upon OVA treatment. **(A)** Number of CD4^+^ T cells in the lungs of PBS-treated or OVA-immunized GFP-C5aR1^fl/fl^ or LysM-C5aR1 KO mice. Values shown are the mean ± SEM; n = 6–8 per group. **(B)** Expression of *FoxP3* (Treg), *GATA3* (Th2), *RORγT* (Th17), and *TBX21* (Th1) transcripts in sorted CD4^+^CD44^+^CD62L^-^ T cells. The abundance of transcripts was evaluated after reverse transcription by real-time PCR. Values shown are the mean abundance of target mRNA as compared to actin; n = 6–8 per group. **(C)** Expression of the Th2 and Th17 cytokines IL-13 and IL-17A, respectively, in sorted CD4^+^CD44^+^CD62L^-^ T cells as determined by real-time PCR. Values shown are the mean abundance of target mRNA as compared to actin; n = 6–8 per group. **(D)** ILC2 cell numbers in the lungs of PBS-treated or OVA-immunized GFP-C5aR1^fl/fl^ or LysM-C5aR1 KO mice. Values shown are the mean ± SEM; n = 6–8 per group. Asterisks indicate significant differences between PBS and OVA treatment groups; * p<0.05, ** p < 0.01, and *** p <0.001.

Next, we assessed the impact of conditional C5aR1 deletion in LysM-expressing cells on T cell recruitment and skewing toward the Th2/Th17 phenotype compared to the GFP-C5aR1^fl/fl^controls. First, we observed a slight increase in total CD4^+^ T cell numbers upon OVA treatment in the lungs of GFP-C5aR1^fl/fl^ mice, which was higher in LysM-C5aR1 KO animals (Fig 4A; left panel).

This increase in CD4^+^ T cells was mainly due to an increase in CD44^+^CD62L^-^ effector T cells (Fig 4A; right panel). By real-time PCR of lineage-related transcription factors *GATA3*, *RORγt*, *TBX21* and *Foxp3*, we confirmed the dominance of Th2/Th17 effector T cell subtypes and the high abundance of Treg cells in CD44^+^CD62L^-^ effector cells from LysM-C5aR1 KO and GFP-C5aR1^fl/fl^ mice (Fig 4B). In line with this finding, we found high and similar abundance of *IL-17A* and *IL-13* transcripts in sorted CD44^+^CD62L^-^ effector T cells from GFP-C5aR1^fl/fl^ and LysM-C5aR1 KO mice (Fig 4C). In addition to CD4^+^ T cells, the number of type 2 innate lymphoid cells (ILC2) increased in the lungs of asthmatic LysM-C5aR1 KO and GFP-C5aR1^fl/fl^ mice following OVA-immunization (Fig 4D). In summary, the absence of C5aR1 in LysM^+^ cells, did not affect the recruitment or the differentiation of Th2/Th17 CD4^+^ effector cells and ILC2 cells.

**Fig 5:**
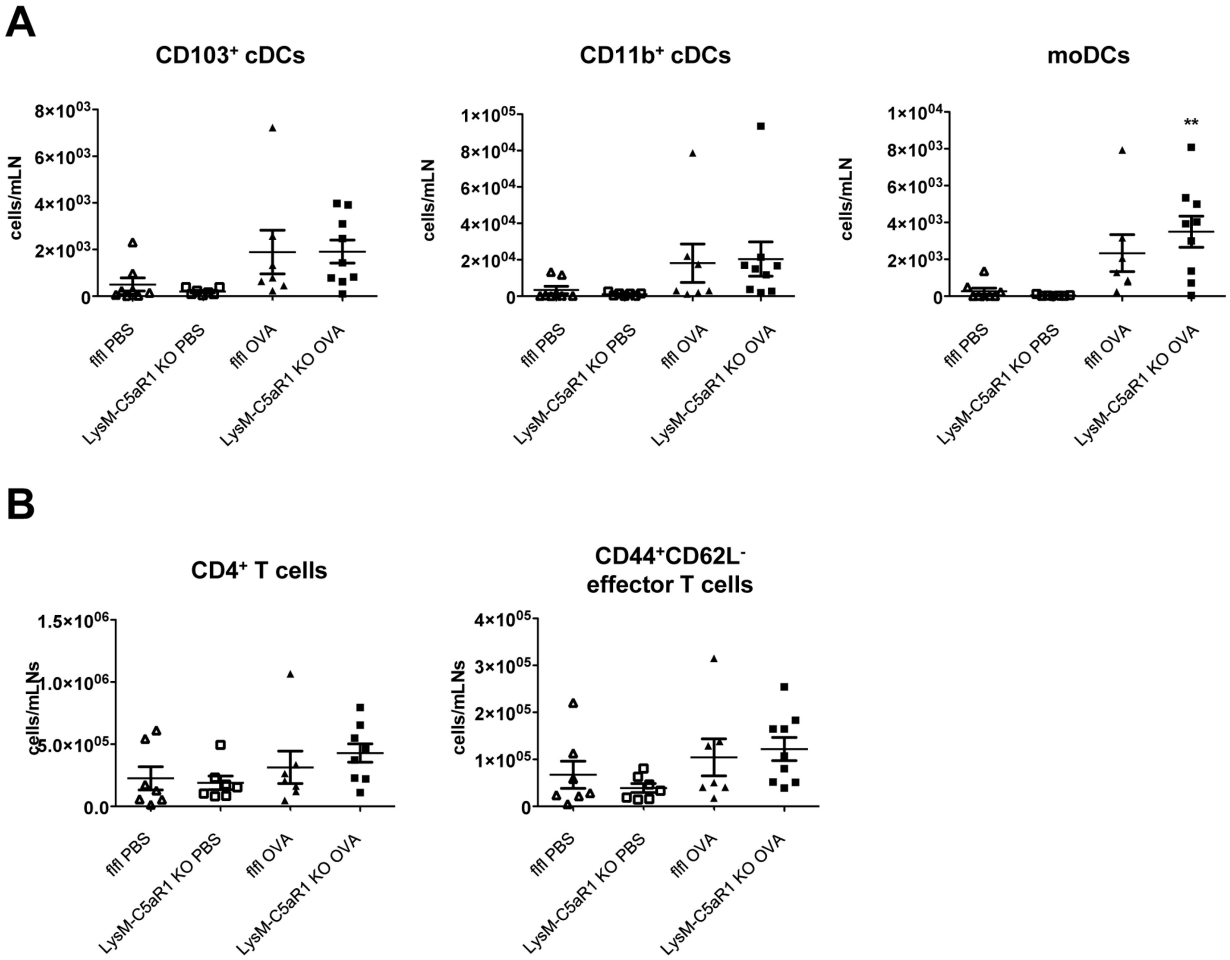
Similar accumulation of DCs and CD4^+^ T cells in mLNs of GFP-C5aR1^fl/fl^ or LysM-C5aR1 KO mice following OVA-immunization. **(A)** Numbers of different DC subsets in mLNs of PBS-treated or OVA-immunized GFP-C5aR1^fl/fl^ or LysM-C5aR1 KO mice. Values shown are the mean ± SEM; n = 7–9 per group. **(B)** Numbers CD4^+^, and CD44^+^CD62L^-^ or CD44^-^CD62L^+^ T cells in mLNs of PBS-treated or OVA-immunized GFP-C5aR1^fl/fl^ or LysM-C5aR1 KO mice. Values shown are the mean ± SEM; n = 7–9 per group. Asterisks indicate significant differences between the PBS and OVA treatment groups; * p<0.05.

Finally, we determined the impact of C5aR1 deletion in LysM-expressing cells on the accumulation of DCs and CD4^+^ T cells in the mediastinal lymph nodes (mLN) after the last OVA challenge. We found a slight increase in CD11b^+^ and CD103^+^ cDCs and moDCs in response to OVA as compared with PBS treatment. However, the DC numbers in GFP-C5aR1^fl/fl^ and LysM-C5aR1 KO mice were similar (Fig 5A).

In line with the increased numbers of DCs upon OVA challenge, we also observed higher numbers of total CD4^+^, CD44^+^CD62L^-^ and CD44^-^CD62L^+^ T cells in the mLNs after OVA challenge, which were comparable in the two strains (Fig 5B).

## Discussion

C5a regulates the development of experimental allergic asthma during allergen sensitization and the effector phase through activation of its two cognate receptors C5aR1 and C5aR2 [1]. The relative importance of C5aR1 expression and activation on defined immune cells of the innate and the adaptive immune system during the course of the disease remains elusive. Recently, we reported the expression of C5aR1 in pulmonary neutrophils, macrophages, CD11b^+^ cDCs and moDCs using a floxed GFP-C5aR1 knock-in mouse [9]. In addition, we demonstrated that the expression of C5aR1 was modulated in eosinophils, AMs and DCs in an OVA-induced allergic asthma model [8]. Breeding the GFP-C5aR1^fl/fl^ reporter mouse to a LysMCre mouse resulted in a LysM-C5aR1 KO, in which C5aR1 was specifically deleted in neutrophils and macrophages [9]. Here, we used these two mouse strains as tools to delineate the impact of C5aR1-specific deletion in LysM-expressing cells on the development of different characteristics of allergic asthma.

LysM has been described in neutrophils and mature macrophages [24], and the conditional deletion of GFP-C5aR1 was shown in BM-neutrophils and peritoneal macrophages [9]. However, alveolar macrophages differ from other tissue macrophages through the strong expression of SiglecF and CD11c [16]. First, we demonstrated that in both, airway and tissue-associated alveolar macrophages GFP-C5aR1 was deleted under steady state conditions and in response to PBS-treatment in LysM-C5aR1 KO mice, confirming that LysM is expressed in alveolar macrophages [25]. In addition, we showed that lung neutrophils show a marked decrease of GFP and C5aR1 expression in LysM-C5aR1 KO mice. This decrease was similar to what we had observed in bone marrow neutrophils. Importantly, C5a failed to induce any increase in intracellular Ca^2+^ in bone marrow neutrophils, confirming efficient deletion of the IRES *C5ar1* cassette. In support of this view, C5aR1 expression was absent in bone marrow neutrophils from LysM-C5aR1 KO mice in Western blot analysis [9]. In contrast, tissue resident eosinophils from LysM-C5aR1 KO mice did not show any reduction of the GFP-C5aR1 signal when compared to the GFP-C5aR1^fl/fl^ controls. The absence of C5aR1 in neutrophils and AMs of LysM-C5aR1 KO mice did not affect their numbers in the airways in response to PBS-treatment. In contrast, the number of airway AMs was higher in the absence of C5aR1 when compared to GFP-C5aR1^fl/fl^ controls. This may indicate that activation of the C5a/C5aR1 axis is an important control mechanism for AM survival in the airways since C5aR1-induced apoptosis of AMs has been described in an acute lung injury model [26]. In addition to AMs, we observed that not only resident moDCs were negative for GFP-C5aR1 but also CD11b^+^ cDCs. Since monocytes express large amounts of lysozyme and can give rise to pulmonary moDCs [27], our observation suggests that the fraction of C5aR1^+^ CD11b^+^ cDCs may originate from monocytes.

We assessed the asthma phenotype in LysM-C5aR1 KO and their GFP-C5aR1^fl/fl^ littermates in an experimental OVA-driven allergic asthma model. Surprisingly, both strains developed a strong but similar allergic phenotype with comparable AHR and mucus production. However, neutrophilic airway inflammation was markedly reduced in asthmatic LysM-C5aR1 KO as compared with GFP-C5aR1^fl/fl^ mice. C5a is a major chemoattractant for neutrophils [28] that can be found in human allergic airways [29, 30] and in mice upon cowshed dust extract administration [31]. These data suggest that the C5a/C5aR1 axis plays a major role in the recruitment of neutrophils into the airways. Surprisingly, the accumulation of neutrophils in the lung tissue is not affected by the absence of C5aR1, suggesting that only the neutrophilic migration from the lung tissue into the airways but not the initial migration into the lung tissue depends on C5a/C5aR1 signaling.

The conditional depletion of C5aR1 in AMs did not change their numbers in the airways or lung tissue under inflammatory conditions. This observation is in line with our earlier report showing that upon allergic asthma inflammation, AMs lose the expression of C5aR1 [8], suggesting that activation of C5aR1 is important for AM function at steady state rather than during the inflammatory process. Further, C5aR1 has been shown to be an important regulator of TLR4 signaling [14, 15] and may regulate tolerogenic programming in resting AMs [32].

The absence of C5aR1 in CD11b^+^ cDCs and moDCs did not result in changes in DC numbers in lung and mLNs, similar to what we observed in adoptive transfer models of allergen-pulsed bone marrow DCs [20]. Among the pulmonary DC subsets, CD11b^+^ cDCs but not moDCs have been reported as the dominant cell type driving Th2 cell differentiation in vivo [16]. Our data show that the conditional depletion of C5aR1 in CD11b^+^ cDCs did not affect the development of Th2/Th17 cells in the OVA model of allergic asthma, similar to what was reported using C5aR1^-/-^ BMDCs [7, 20]. These data support the idea that C5aR1 is mainly expressed in DCs of monocytic origin, which regulate proliferation and differentiation of Th cells only at high antigen concentrations [16].

Furthermore, our data suggest that the expression of C5aR1 in eosinophils might be more important than previously thought. Since C5aR1 is still expressed in LysM-C5aR1 KO eosinophils, our data suggest a role of eosinophilic C5aR1 in the development and/or severity of the allergic asthma phenotype. This is supported by the observations that upon allergic asthma conditions, the expression of C5aR1 is increased in eosinophils [8]. The availability of eosinophil-specific Cre deleter mice [33] will help to delineate the role of the C5a/C5aR1 axis in these cells for the development of allergic asthma.

Collectively, we demonstrate that C5aR1 is specifically deleted in pulmonary neutrophils, macrophages and moDCs in LysM-C5aR1 KO mice. We show that this mouse strain proves useful to determine the impact of C5aR1 on the pulmonary recruitment of LysM-expressing cells and the development of allergic asthma. While the absence of C5aR1 impaired the recruitment of neutrophils from the lung into the airway, this decreased recruitment did not affect the allergic phenotype. Our findings further suggest that the C5a/C5aR1 axis plays a surprisingly minor direct role for the recruitment of neutrophils, macrophages and moDCs into the lung tissue. Finally, we demonstrate that C5aR1 activation of LysM-expressing cells does not contribute to the development and severity of allergic asthma. Future studies using Cre deleter rmice that will allow specific deletion of C5aR1 in bona fide cDCs, eosinophils, basophils, or mast cells will help to broaden our understanding of how the C5a/C5aR1 axis regulates allergic asthma development.

## Abbreviations

AHR: airway hyperresponsiveness
AT: anaphylatoxin
BAL: bronchoalveolar lavage fluid
C3a: complement 3a
C5a: complement 5a
C5aR1: C5a receptor 1
cDCs: conventional dendritic cells
fl: flox
MFI: mean fluorescence intensity
mLN: mediastinal lymph node
moDCs: monocyte-derived dendritic cells
OVA: ovalbumin
UTR: untranslated region
WT: wild type

## References

[1] Laumonnier Y, Wiese AV, Figge J, Karsten C. Regulation and function of anaphylatoxins and their receptors in allergic asthma. Mol Immunol. 2017;84:51–6. Epub 2016/12/06. doi: 10.1016/j.molimm.2016.11.013 . 27916272 10.1016/j.molimm.2016.11.013

[2] Kohl J, Baelder R, Lewkowich IP, Pandey MK, Hawlisch H, Wang L, et al A regulatory role for the C5a anaphylatoxin in type 2 immunity in asthma. J Clin Invest. 2006;116(3):783–96. Epub 2006/03/03. doi: 10.1172/JCI26582 16511606 10.1172/JCI26582 PMC1386108

[3] Staab EB, Sanderson SD, Wells SM, Poole JA. Treatment with the C5a receptor/CD88 antagonist PMX205 reduces inflammation in a murine model of allergic asthma. Int Immunopharmacol. 2014;21(2):293–300. Epub 2014/05/27. doi: 10.1016/j.intimp.2014.05.008 24859057 10.1016/j.intimp.2014.05.008 PMC4108528

[4] Lajoie S, Lewkowich IP, Suzuki Y, Clark JR, Sproles AA, Dienger K, et al Complement-mediated regulation of the IL-17A axis is a central genetic determinant of the severity of experimental allergic asthma. Nat Immunol. 2010;11(10):928–35. Epub 2010/08/31. doi: 10.1038/ni.1926 20802484 10.1038/ni.1926 PMC2943538

[5] Weaver DJ Jr., Reis ES, Pandey MK, Kohl G, Harris N, Gerard C, et al C5a receptor-deficient dendritic cells promote induction of Treg and Th17 cells. Eur J Immunol. 2010;40(3):710–21. Epub 2009/12/18. doi: 10.1002/eji.200939333 20017191 10.1002/eji.200939333 PMC3040298

[6] Zhang X, Lewkowich IP, Kohl G, Clark JR, Wills-Karp M, Kohl J. A protective role for C5a in the development of allergic asthma associated with altered levels of B7-H1 and B7-DC on plasmacytoid dendritic cells. J Immunol. 2009;182(8):5123–30. Epub 2009/04/04. doi: 10.4049/jimmunol.0804276 19342693 10.4049/jimmunol.0804276 PMC2923383

[7] Schmudde I, Strover HA, Vollbrandt T, Konig P, Karsten CM, Laumonnier Y, et al C5a receptor signalling in dendritic cells controls the development of maladaptive Th2 and Th17 immunity in experimental allergic asthma. Mucosal Immunol. 2013;6(4):807–25. Epub 2012/12/06. doi: 10.1038/mi.2012.119 . 23212198 10.1038/mi.2012.119

[8] Ender F, Wiese AV, Schmudde I, Sun J, Vollbrandt T, Konig P, et al Differential regulation of C5a receptor 1 in innate immune cells during the allergic asthma effector phase. PLoS One. 2017;12(2):e0172446 Epub 2017/02/24. doi: 10.1371/journal.pone.0172446 28231307 10.1371/journal.pone.0172446 PMC5322932

[9] Karsten CM, Laumonnier Y, Eurich B, Ender F, Broker K, Roy S, et al Monitoring and cell-specific deletion of C5aR1 using a novel floxed GFP-C5aR1 reporter knock-in mouse. J Immunol. 2015;194(4):1841–55. Epub 2015/01/16. doi: 10.4049/jimmunol.1401401 . 25589074 10.4049/jimmunol.1401401

[10] Goetzl EJ. Modulation of human eosinophil polymorphonuclear leukocyte migration and function. Am J Pathol. 1976;85(2):419–36. Epub 1976/11/01. 793410 PMC2032558

[11] Elsner J, Oppermann M, Kapp A. Detection of C5a receptors on human eosinophils and inhibition of eosinophil effector functions by anti-C5a receptor (CD88) antibodies. Eur J Immunol. 1996;26(7):1560–4. Epub 1996/07/01. doi: 10.1002/eji.1830260723 . 8766561 10.1002/eji.1830260723

[12] Fujiu T, Kato M, Kimura H, Tachibana A, Suzuki M, Nako Y, et al Cellular adhesion is required for effector functions of human eosinophils via G-protein coupled receptors. Ann Allergy Asthma Immunol. 2002;89(1):90–8. Epub 2002/07/27. doi: 10.1016/S1081-1206(10)61917-5 . 12141728 10.1016/S1081-1206(10)61917-5

[13] Haggadone MD, Grailer JJ, Fattahi F, Zetoune FS, Ward PA. Bidirectional Crosstalk between C5a Receptors and the NLRP3 Inflammasome in Macrophages and Monocytes. Mediators Inflamm. 2016;2016:1340156 Epub 2016/07/07. doi: 10.1155/2016/1340156 27382187 10.1155/2016/1340156 PMC4921141

[14] Hashimoto M, Hirota K, Yoshitomi H, Maeda S, Teradaira S, Akizuki S, et al Complement drives Th17 cell differentiation and triggers autoimmune arthritis. J Exp Med. 2010;207(6):1135–43. Epub 2010/05/12. doi: 10.1084/jem.20092301 20457757 10.1084/jem.20092301 PMC2882841

[15] Hawlisch H, Belkaid Y, Baelder R, Hildeman D, Gerard C, Kohl J. C5a negatively regulates toll-like receptor 4-induced immune responses. Immunity. 2005;22(4):415–26. Epub 2005/04/23. doi: 10.1016/j.immuni.2005.02.006 . 15845447 10.1016/j.immuni.2005.02.006

[16] Plantinga M, Guilliams M, Vanheerswynghels M, Deswarte K, Branco-Madeira F, Toussaint W, et al Conventional and monocyte-derived CD11b(+) dendritic cells initiate and maintain T helper 2 cell-mediated immunity to house dust mite allergen. Immunity. 2013;38(2):322–35. Epub 2013/01/29. doi: 10.1016/j.immuni.2012.10.016 . 23352232 10.1016/j.immuni.2012.10.016

[17] Quell KM, Karsten CM, Kordowski A, Almeida LN, Briukhovetska D, Wiese AV, et al Monitoring C3aR Expression Using a Floxed tdTomato-C3aR Reporter Knock-in Mouse. J Immunol. 2017;199(2):688–706. Epub 2017/06/20. doi: 10.4049/jimmunol.1700318 . 28626064 10.4049/jimmunol.1700318

[18] Skokowa J, Ali SR, Felda O, Kumar V, Konrad S, Shushakova N, et al Macrophages induce the inflammatory response in the pulmonary Arthus reaction through G alpha i2 activation that controls C5aR and Fc receptor cooperation. J Immunol. 2005;174(5):3041–50. Epub 2005/02/25. . 15728518 10.4049/jimmunol.174.5.3041

[19] Verschoor A, Karsten CM, Broadley SP, Laumonnier Y, Kohl J. Old dogs-new tricks: immunoregulatory properties of C3 and C5 cleavage fragments. Immunol Rev. 2016;274(1):112–26. Epub 2016/10/27. doi: 10.1111/imr.12473 . 27782330 10.1111/imr.12473

[20] Engelke C, Wiese AV, Schmudde I, Ender F, Strover HA, Vollbrandt T, et al Distinct roles of the anaphylatoxins C3a and C5a in dendritic cell-mediated allergic asthma. J Immunol. 2014;193(11):5387–401. Epub 2014/10/31. doi: 10.4049/jimmunol.1400080 . 25355927 10.4049/jimmunol.1400080

[21] Monticelli LA, Sonnenberg GF, Abt MC, Alenghat T, Ziegler CG, Doering TA, et al Innate lymphoid cells promote lung-tissue homeostasis after infection with influenza virus. Nat Immunol. 2011;12(11):1045–54. Epub 2011/09/29. doi: 10.1031/ni.2131 21946417 10.1031/ni.2131 PMC3320042

[22] Zhang X, Schmudde I, Laumonnier Y, Pandey MK, Clark JR, Konig P, et al A critical role for C5L2 in the pathogenesis of experimental allergic asthma. J Immunol. 2010;185(11):6741–52. doi: 10.4049/jimmunol.1000892 . 20974988 10.4049/jimmunol.1000892

[23] Clausen BE, Burkhardt C, Reith W, Renkawitz R, Forster I. Conditional gene targeting in macrophages and granulocytes using LysMcre mice. Transgenic Res. 1999;8(4):265–77. Epub 2000/01/06. . 10621974 10.1023/a:1008942828960

[24] Cross M, Mangelsdorf I, Wedel A, Renkawitz R. Mouse lysozyme M gene: isolation, characterization, and expression studies. Proc Natl Acad Sci U S A. 1988;85(17):6232–6. Epub 1988/09/01. 3413093 10.1073/pnas.85.17.6232 PMC281943

[25] Nieuwenhuizen NE, Kirstein F, Jayakumar J, Emedi B, Hurdayal R, Horsnell WG, et al Allergic airway disease is unaffected by the absence of IL-4Ralpha-dependent alternatively activated macrophages. J Allergy Clin Immunol. 2012;130(3):743–50 e8. Epub 2012/05/04. doi: 10.1016/j.jaci.2012.03.011 . 22552110 10.1016/j.jaci.2012.03.011

[26] Hu R, Chen ZF, Yan J, Li QF, Huang Y, Xu H, et al Complement C5a exacerbates acute lung injury induced through autophagy-mediated alveolar macrophage apoptosis. Cell Death Dis. 2014;5:e1330 Epub 2014/07/18. doi: 10.1038/cddis.2014.274 25032853 10.1038/cddis.2014.274 PMC4123068

[27] Varol C, Landsman L, Fogg DK, Greenshtein L, Gildor B, Margalit R, et al Monocytes give rise to mucosal, but not splenic, conventional dendritic cells. J Exp Med. 2007;204(1):171–80. Epub 2006/12/28. doi: 10.1084/jem.20061011 17190836 10.1084/jem.20061011 PMC2118434

[28] Klos A, Tenner AJ, Johswich KO, Ager RR, Reis ES, Kohl J. The role of the anaphylatoxins in health and disease. Mol Immunol. 2009;46(14):2753–66. Epub 2009/05/30. doi: 10.1016/j.molimm.2009.04.027 19477527 10.1016/j.molimm.2009.04.027 PMC2725201

[29] Krug N, Tschernig T, Erpenbeck VJ, Hohlfeld JM, Kohl J. Complement factors C3a and C5a are increased in bronchoalveolar lavage fluid after segmental allergen provocation in subjects with asthma. Am J Respir Crit Care Med. 2001;164(10 Pt 1):1841–3. Epub 2001/12/06. doi: 10.1164/ajrccm.164.10.2010096 . 11734433 10.1164/ajrccm.164.10.2010096

[30] Marc MM, Korosec P, Kosnik M, Kern I, Flezar M, Suskovic S, et al Complement factors c3a, c4a, and c5a in chronic obstructive pulmonary disease and asthma. Am J Respir Cell Mol Biol. 2004;31(2):216–9. Epub 2004/03/25. doi: 10.1165/rcmb.2003-0394OC . 15039137 10.1165/rcmb.2003-0394OC

[31] Stiehm M, Peters K, Wiesmuller KH, Bufe A, Peters M. A novel synthetic lipopeptide is allergy-protective by the induction of LPS-tolerance. Clin Exp Allergy. 2013;43(7):785–97. Epub 2013/06/22. doi: 10.1111/cea.12116 . 23786285 10.1111/cea.12116

[32] Hussell T, Bell TJ. Alveolar macrophages: plasticity in a tissue-specific context. Nat Rev Immunol. 2014;14(2):81–93. Epub 2014/01/22. doi: 10.1038/nri3600 . 24445666 10.1038/nri3600

[33] Doyle AD, Jacobsen EA, Ochkur SI, Willetts L, Shim K, Neely J, et al Homologous recombination into the eosinophil peroxidase locus generates a strain of mice expressing Cre recombinase exclusively in eosinophils. J Leukoc Biol. 2013;94(1):17–24. Epub 2013/05/01. doi: 10.1189/jlb.0213089 23630390 10.1189/jlb.0213089 PMC3685019

